# Supplementary material for: Gene inversion potentiates bacterial evolvability and virulence
Source: Nat Commun. 2018 Nov 7;9:4662. doi: 10.1038/s41467-018-07110-3 (PMC6220195; doi:10.1038/s41467-018-07110-3)
Supplement: Supplementary file 1 — Supplementary Information [file 41467_2018_7110_MOESM1_ESM.pdf]

Gene inversion potentiates bacterial evolvability and virulence

Merrikh et al., Nature Communications, 2018

Supplemental Data Tables

	Species	Abbreviation	% GC	*All Gene Orientation Bias*	*Core Genes (Used in TimeZone Analysis)*		Gene Inversion Data					
							All CD Genes			All HO Genes		
				% CD Genes	# CD	# HO	Total #	# Flipped	% Flipped	Total #	# Flipped	% Flipped
Gram +	*M. gallisepticum*	*Mg*	31	80%	340	95	651	166	25%	159	121	76%
	S. aureus	*Sa*	32	77%	1461	466	2216	84	4%	656	428	65%
	B. subtilis	*Bs*	44	73%	2463	861	3104	127	4%	1139	777	68%
Gram -	*C. jejuni*	*Cj*	31	62%	826	548	1048	23	2%	651	529	81%
	K. pneumoniae	*Kp*	57	55%	2045	1744	2999	465	16%	2405	1102	46%
--	*M. tuberculosis*	*Mtb*	66	59%	917	675	2415	603	25%	1665	712	43%

Supplementary Table 1. Genome analysis data. Species naming abbreviations used in the main figures are presented, as well as GC content (% GC), the number of core genes identified by TimeZone during retained mutation analysis binned by orientation, and the number of genes identified as inverted based upon GC skew analysis (genes with negative average GC skew values).

		% HO Genes in Genome (HO/Total)				
		**Core Genes**		**All Genes**		**Ratio**
Gram +	*Mycoplasma gallisepticum*	22%	÷	20%	=	1.11
	Staphylococcus aureus	24%	÷	23%	=	1.06
	Bacillus subtilis	26%	÷	27%	=	0.96
Gram -	*Campylobacter jejuni*	39%	÷	38%	=	1.03
	Klebsiella pneumoniae	46%	÷	45%	=	1.03
N/A	*Mycobacterium tuberculosis*	42%	÷	41%	=	1.04
	Average:					1.04
	Standard Error of the Mean:					0.02

Supplementary Table 2. Comparison of the head-on gene content among all genes in the genome, or among highly conserved genes identified as core genes. A comparison of the two percentages are presented as a ratio, showing that head-on genes are just as likely to be highly conserved (core) as co-directional genes. These results are highly consistent across species.

Species	Gene Orientation	Gene	Strand	Location	Function	dN/dS
K. pneumoniae	HO	KPHS_41220	+	4154244-4154390	hypothetical protein	2.14068
		KPHS_07770	-	822865-823032	hypothetical protein	2.09719
		KPHS_49520	+	4958413-4958772	hypothetical protein	1.91704
		KPHS_20390	-	2097641-2097802	hypothetical protein	1.91158
		KPHS_16650	-	1723634-1723891	hypothetical protein	1.90048
		KPHS_46690	+	4691144-4691284	hypothetical protein	1.84225
		KPHS_35140	+	3512446-3512571	hypothetical protein	1.45085
		KPHS_10210	-	1080457-1080591	putative LysR-family transcriptional regulator	1.44044
		KPHS_08540	-	905073-905657	hypothetical protein	1.29234
		KPHS_43320	+	4369597-4369725	hypothetical protein	1.27967
		KPHS_46260	+	4650646-4650828	hypothetical protein	1.13584
		KPHS_34250	+	3400969-3401145	hypothetical protein	1.0898
		KPHS_14080	-	1461213-1461347	hypothetical protein	1.08617
		KPHS_15850	-	1649903-1650049	hypothetical protein	1.03738
		KPHS_15090	-	1568301-1568927	cobC, a phosphohistidine protein	1.03577
		KPHS_35710	+	3578064-3578183	hypothetical protein	1.01247
		KPHS_12280	-	1283596-1283931	hypothetical protein	1.00419
	CD	KPHS_10920	+	1152251-1152415	hypothetical protein	3.34718
		KPHS_17050	+	1770534-1770659	hypothetical protein	2.63325
		KPHS_41950	-	4224556-4224726	hypothetical protein	2.34803
		KPHS_38430	-	3875663-3875812	hypothetical protein	2.18385
		KPHS_33160	-	3300097-3300546	hypothetical protein	2.18355
		KPHS_41380	-	4166238-4166393	hypothetical protein	1.95461
		KPHS_49150	-	4910702-4911127	hypothetical protein	1.83428
		KPHS_51790	-	5198672-5198782	hypothetical protein	1.4331
		KPHS_37280	-	3762205-3762960	2-succinyl-6-hydroxy-2,4-cyclohexadiene-1-carboxylante synthase	1.43009
		KPHS_46320	-	4656947-4657060	hypothetical protein	1.22911
		KPHS_45940	-	4618512-4618655	short-chain dehydrogenase	1.19156
		KPHS_26960	-	2706948-2707163	hypothetical protein	1.18343
		KPHS_43710	-	4408166-4408585	arsenate reductase	1.1637
		KPHS_52520	-	5271244-5271375	hypothetical protein	1.14351
		KPHS_14230	+	1476352-1476462	hypothetical protein	1.03409
M. tuberculosis	HO	*Rv1458c*	-	1643322-1644260	ABC transporter, confers resistance to multiple front line antbiotics	2.71873
		Rv1587c	-	1788165-1789163	hypothetical protein	2.31217
		PE_PGRS32	-	2044926-2046842	PE-PGRS family protein PE_PGRS32	2.12084
		cycA	-	1929789-1931456	D-serine/alanine/glycine transporter proteinCycA	1.78872
		Rv2891	+	3200266-3201012	hypothetical protein	1.78523
		PE_PGRS19	-	1188424-1190424	PE-PGRS family protein PE_PGRS19	1.77354
		Rv3359	+	3771344-3772531	oxidoreductase	1.64695
		glgB	-	1490120-1492312	1,4-alpha-glucan branching protein	1.62527
		ndhA	-	471230-472639	NADH dehydrogenase NdhA	1.56736
		Rv1674c	-	1899263-1899916	transcriptional regulator. dN/dS = 1.5	1.54021
		PE1	-	177546-179309	PE family protein PE1	1.46225
		Rv2567	+	2889795-2892446	hypothetical protein	1.46057
		mce3F	+	2215257-2216567	Mce family protein Mce3F	1.28277
		Rv0493c	-	583704-584690	hypothetical protein	1.2686
		PE_PGRS52	+	3801653-3803845	PE-PGRS family protein PE_PGRS52	1.20202
		PE_PGRS30	-	1862350-1865382	PE-PGRS family protein PE_PGRS30	1.19185
		pknH	-	1413963-1415840	serine/threonine-protein kinase PknH	1.17944
		kasA	+	2518115-2519362	3-oxoacyl-ACP synthase 1	1.15835
		aftA	+	4237932-4239860	arabinofuranosyltransferase	1.15532
		pknA	-	17470-18762	serine/threonine-protein kinase PknA	1.1521
		ctpH	-	510705-515321	metal cation transporting ATPase H	1.13981
		Rv2090	+	2347373-2348551	5'-3' exonuclease	1.13919
		Rv0193c	-	224727-226571	hypothetical protein	1.13627
		Rv0376c	-	453233-454372	hypothetical protein	1.11957
		cut3	+	3872617-3873402	cutinase	1.11806
		embC	+	4239863-4243144	essential gene, arabinosyltransferase C	1.114
		helZ	+	2360240-2363278	helicase HelZ	1.07175
		Rv0610c	-	704755-705909	INVERTER. hypothetical protein	1.06925
		Rv1320c	-	1482517-1484217	adenylate cyclase	1.05978
		ppsB	+	3251072-3255685	phthiocerol synthesis polyketide synthase type IPpsB	1.05071
		fadE35	+	4251085-4252863	acyl-CoA dehydrogenase FadE35	1.04224
		PPE59	+	3847165-3847698	PPE family protein PPE59	1.03844
		Rv3263	+	3643177-3644835	DNA methylase	1.03663
		Rv3254	+	3633675-3635060	hypothetical protein	1.02206

	CD	*PE_PGRS5*	+	361334-363106	PE-PGRS family protein PE_PGRS5	3.18914
		pks8	+	1881704-1886509	polyketide synthase	2.5534
		cyp138	+	163366-164688	cytochrome P450 Cyp138	2.43081
		ubiA	-	4268928-4269833	decaprenyl-phosphate phosphoribosyltransferase	2.2167
		metH	-	2382492-2386067	methionine synthase	2.09173
		ligD	+	1046136-1048412	multifunctional non-homologous end joining DNA	2.05833
		PPE3	+	339364-340971	PPE family protein PPE3	1.92037
		Rv1999c	-	2243819-2245138	transporter	1.90122
		glgP	+	1494564-1497152	glycogen phosphorylase	1.61697
		PE_PGRS49	-	3736987-3738000	PE-PGRS family protein PE_PGRS49	1.57194
		mgtC	+	2053443-2054144	Mg2+ transport P-type ATPase MgtC	1.52366
		Rv0064	+	68620-71556	transmembrane protein	1.52079
		Rv0797	+	890388-891479	insertion sequence element IS1547 transposase	1.43357
		dop	-	2370908-2372569	pup deamidase/depupylase	1.37333
		pks6	+	485731-489936	membrane bound polyketide synthase	1.37156
		Rv2177c	-	2439285-2439947	transposase	1.34233
		gyrA	+	7302-9815	DNA gyrase subunit A	1.30515
		gyrA	+	7302-9815	DNA gyrase subunit A	1.30515
		atsD	+	756137-758497	arylsulfatase AtsD	1.28377
		mqo	-	3160583-3162061	malate:quinone oxidoreductase	1.18949
		lpqI	+	287186-288349	lipoprotein LpqI	1.16498
		eccA2	-	4364982-4366838	ESX-2 secretion system protein EccA	1.16271
		fadD30	+	483977-485731	long-chain-fatty-acid--AMP ligase FadD30	1.15979
		Rv0818	+	910972-911736	transcriptional regulator	1.15505
		mkl	+	751517-752593	ABC transporter ATP-binding protein	1.14454
		phoR	+	852396-853850	two component system response sensor kinasePhoR	1.12098
		Rv1278	+	1427414-1430038	hypothetical protein	1.09582
		ftsK	-	3059858-3062506	DNA translocase FtsK	1.08264
		fadE17	-	2184962-2186188	acyl-CoA dehydrogenase FadE17	1.07862
		eccE1	-	4362035-4363420	ESX-1 secretion system protein EccE1	1.0729
		Rv3193c	-	3560197-3563172	transmembrane protein	1.06891
		PE_PGRS46	-	2960108-2962441	PE-PGRS family protein PE_PGRS46	1.03515
		secA2	+	2066457-2068880	accessory Sec system translocase SecA2	1.03394
		Rv0859	+	955077-956285	acyltransferase	1.02366
		fadD25	+	1712302-1714050	fatty-acid--CoA ligase FadD25	1.01953
		Rv0221	+	264067-265473	diacylglycerol O-acyltransferase	1.0173
M. gallisepticum	HO	*MGA_RS04080*	+	963040-963393	hypothetical protein	2.44786
		MGA_RS01705	-	409201-410535	signal recognition particle protein	1.92437
		MGA_RS03855	+	963407-963790	aldehyde dehydrogenase	1.3198
		MGA_RS00830	-	187910-188581	methionyl-tRNA synthetase	1.07682
	CD	*MGA_RS03885*	-	969836-970087	hypothetical protein	1.31604
		MGA_RS00845	+	189840-191027	hypothetical protein	1.31296
		MGA_RS03840	-	958788-961193	hypothetical protein	1.06655
S. aureus	HO	*SAOUHSC_01897*	+	1808335-1808802	hypothetical protein, possibly RNA polymerase sigma factor sigS	1.98636
		SAOUHSC_01376	-	1321117-1321587	hypothetical protein	1.82282
		SAOUHSC_00193	-	213827-213931	hypothetical protein	1.42735
		SAOUHSC_02780	+	2555609-2555791	hypothetical protein	1.36884
		SAOUHSC_02973	+	2735412-2735867	hypothetical protein	1.34441
		SAOUHSC_02890	+	2663260-2663394	hypothetical protein	1.29276
		SAOUHSC_01353	-	1296730-1296825	hypothetical protein	1.15446
		SAOUHSC_01181	-	1133140-1133415	hypothetical protein	1.09018
		SAOUHSC_00376	-	383361-383894	hypothetical protein	1.06534
		SAOUHSC_01489	+	1445100-1445411	hypothetical protein	1.06421
	CD	*SAOUHSC_01298*	+	1249705-1249824	hypothetical protein	1.87526
		SAOUHSC_A01436	-	1450029-1450217	hypothetical protein	1.80689
		SAOUHSC_A02811	-	2731691-2731855	hypothetical protein	1.72183
		SAOUHSC_02171	-	2035678-2036166	staphylokinase	1.5283
		SAOUHSC_03041	-	2811511-2812005	hypothetical protein	1.35512
		SAOUHSC_01109	+	1070176-1070340	hypothetical protein	1.31058
		SAOUHSC_02634	-	2421664-2421789	hypothetical protein	1.29002
		SAOUHSC_02957	-	2719289-2719486	hypothetical protein	1.27815
		SAOUHSC_01603	-	1528899-1529150	hypothetical protein	1.24612
		SAOUHSC_01841	-	1747199-1747288	hypothetical protein	1.17382
		SAOUHSC_02717	-	2498233-2498373	hypothetical protein	1.11092
		SAOUHSC_01260	+	1214102-1214677	CDP-diacylglycerol--glycerol-3-phosphate3-phosphatidyltransferase	1.07642
		SAOUHSC_00491	+	489065-489538	2-amino-4-hydroxy-6-hydroxymethyldihydropteridine pyrophosphokinase	1.02009
		SAOUHSC_01289	+	1245527-1245748	hypothetical protein	1.00676

B.subtilis	HO	antE	+	2602979-2603272	possible sporulation protein, AntE	3.25
		yqjU	+	2467971-2468159	hypothetical protein	1.48
		ypzC	+	2433137-2433370	hypothetical protein	1.39
		yheF	-	1049804-1049926	hypothetical protein	1.19
		ynzK	-	1904396-1904749	hypothetical protein	1.05
	CD	yflD	+	844097-844231	hypothetical protein	1.66129
		ybzI	+	222971-223231	hypothetical protein	1.60677
		yesK	+	757676-757975	hypothetical protein	1.35459
		yngHB	-	1952948-1953166	acetyl-CoA carboxylase biotin carboxyl carrierprotein subunit	1.23238
		ykuN	+	1487038-1487511	flavodoxin	1.18383
		ylxL	+	1717286-1717786	swarming motility protein SwrB	1.06603
C. jejuni	HO	Cj1225	+	1153761-1154006	hypothetical protein	1.02776
	CD	Cj0494	+	460364-460510	exporting protein	1.36064
		Cj1656c	-	1579780-1579959	hypothetical protein	1.33105

Supplementary Table 3. Genes with a dN/dS value exceeding a value of 1, an important indicator of positive selection.

	CD				HO				P-value
	Avg	STDEV	SEM	Total	Avg	STDEV	SEM	Total	
With dN/dS >1	0.143771398	0.002064782	0.002065	15503	0.166575	0.003003	0.003003	9620	<0.0001
Without dN/dS >1	0.116871315	0.001156723	0.001157	15213	0.130272	0.001599	0.001599	9386	<0.0001

Supplementary Table 4. The effect of dN/dS >1 genes on the average dN/dS ratio of all six species. Even after manually removing the data points for genes under overt positive selection (dN/dS >1), the average dN/dS ratio of head-on genes still exceeds that of co-directional genes. These data are consistent with the possibility that positive selection acts on a broad array of head-on genes. *p*-values were calculated using the Z-test (2-tailed). "Total" indicates the number of genes in each category. SEM = Standard error of the mean, STDEV = standard deviation.

Species	Gene Name	Function
M. tuberculosis	*Rv1458c*	antibiotic ABC transporter ATP-binding protein
	pks5	polyketide synthase
	ndhA	NADH dehydrogenase NdhA
	mce3F	Virulence gene. Mce family protein Mce3F
	Rv1320c	Virulence gene. Adenylate cyclase
	narL	nitrate/nitrite response transcriptionalregulator NarL
	espR	Virulence regulator. ESX-1 transcriptional regulator EspR
	espE	ESX-1 secretion-associated protein EspE
	Rv3736	Virulence gene. AraC/XylS family transcriptional regulator
	emrB	multidrug resistance protein EmrB
	mrp	multiple resistance/pH adaptation protein
	Rv1258c	multidrug-efflux transporter
	mmr	multidrug resistance protein Mmr
	PE_PGRS24	PE-PGRS family protein PE_PGRS24
	PE_PGRS25	PE-PGRS family protein PE_PGRS25
	PE_PGRS26	PE-PGRS family protein PE_PGRS26
	PE_PGRS27	PE-PGRS family protein PE_PGRS27
	PE_PGRS28	PE-PGRS family protein PE_PGRS28
	PE_PGRS29	PE-PGRS family protein PE_PGRS29
	PE_PGRS30	PE-PGRS family protein PE_PGRS30
	PE_PGRS32	PE-PGRS family protein PE_PGRS32
	PE_PGRS33	PE-PGRS family protein PE_PGRS33
	PE_PGRS35	PE-PGRS family protein PE_PGRS35
	PE_PGRS4	PE-PGRS family protein PE_PGRS4
	PE_PGRS40	PE-PGRS family protein PE_PGRS40
	PE_PGRS41	acid and phagosome regulated protein AprC
	PE_PGRS44	PE-PGRS family protein PE_PGRS44
	PE_PGRS47	PE-PGRS family protein PE_PGRS47
	PE_PGRS48	PE-PGRS family protein PE_PGRS48
	PE_PGRS51	PE-PGRS family protein PE_PGRS51
	PE_PGRS52	PE-PGRS family protein PE_PGRS52
	PE_PGRS53	PE-PGRS family protein PE_PGRS53
	PE_PGRS54	PE-PGRS family protein PE_PGRS54
	PE_PGRS55	PE-PGRS family protein PE_PGRS55
	PE_PGRS56	PE-PGRS family protein PE_PGRS56
	PE_PGRS57	PE-PGRS family protein PE_PGRS57
	PE_PGRS60	PE-PGRS family-related protein PE_PGRS60
	PE_PGRS61	PE-PGRS family-related protein PE_PGRS61
	PE_PGRS62	PE-PGRS family protein PE_PGRS62
	PE_PGRS7	PE-PGRS family protein PE_PGRS7
	PPE10	PPE family protein PPE10
	PPE12	PPE family protein PPE12
	PPE13	PPE family protein PPE13
	PPE14	PPE family protein PPE14
	PPE15	PPE family protein PPE15
	PPE16	PPE family protein PPE16
	PPE17	PPE family protein PPE17
	PPE19	PPE family protein PPE19
	PPE2	PPE family protein PPE2
	PPE21	PPE family protein PPE21
	PPE22	PPE family protein PPE22
	PPE23	PPE family protein PPE23
	PPE24	PPE family protein PPE24
	PPE36	PPE family protein PPE36
	PPE37	PPE family protein PPE37
	PPE42	PPE family protein PPE42
	PPE5	PPE family protein PPE5
	PPE50	PPE family protein PPE50
	PPE51	PPE family protein PPE51
	PPE57	PPE family protein PPE57
	PPE58	PPE family protein PPE58
	PPE59	PPE family protein PPE59
	PPE6	PPE family protein PPE6
	PPE60	PE family protein PPE60
	PPE61	PPE family protein PPE61
	PPE63	PPE family protein PPE63
	PPE64	PPE family protein PPE64
	PPE68	PPE family protein PPE68
	PPE7	PPE family protein PPE7
	PPE8	PPE family protein PPE8

K. pneumoniae	*KPHS_15380*	Virulence gene. Ferric uptake regulator
	KPHS_34420	positive regulator for ctr capsule biosynthesis, positive transcription factor
	KPHS_34710	pesticin/yersiniabactin TonB-dependent receptor
	KPHS_21500	catecholate siderophore receptor Fiu
	KPHS_21440	Virulence gene. TonB dependent outer membrane siderophore receptor protein
	KPHS_34680	Virulence gene. irp3 protein, yersiniabactin siderophore biosynthetic protein
	KPHS_34700	Virulenge gene. yersiniabactin siderophore biosynthetic protein
	KPHS_05940	putative multidrug ABC transporter ATPase and permease
	KPHS_06130	multidrug resistance protein MdtM
	KPHS_19540	multidrug resistance protein MdtH
	KPHS_23870	putative multidrug resistance efflux pump
	KPHS_27270	multidrug resistance protein membrane translocase
	KPHS_29630	putative multidrug resistance protein
	KPHS_29820	multidrug efflux protein
	KPHS_35860	multidrug efflux system subunit MdtA
	KPHS_35870	multidrug efflux system subunit MdtB
	KPHS_35880	multidrug efflux system subunit MdtC
	KPHS_35890	multidrug efflux system protein MdtE
	KPHS_38740	aminoglycoside/multidrug efflux system
	KPHS_40890	multidrug resistance secretion protein
	KPHS_40900	multidrug resistance protein membrane translocase
	KPHS_43660	multidrug resistance protein MdtN
	KPHS_43670	multidrug efflux system protein MdtO
	KPHS_48270	inner membrane multidrug efflux protein BpeB
	KPHS_52090	multidrug efflux permease EefB
	KPHS_52100	multidrug efflux transport outer membrane protein EefC
	KPHS_52500	multidrug MFS integral membrane transporter
S. aureus	*mecA*	Penicillin binding protein. Mutations confer resistance to penicillin/methicillin.
	agrA	Virulence gene, regulator of RNAIII
	traP	Master virulence regulator, regulates RNAIII
	SA1633	Beta lactamase, part of vSaβ pathogenicity island
	spA	Immune evasion protein, binds Fc portion of host IgG
	sirABC, SAOUHSC_00070-72	Iron siderophore proteins
	SAOUHSC_02696	Methicillin resistance determinant protein FmhA
	SAOUHSC_02609	Fosfomycin resistance protein FosB
	SAOUHSC_01903	Camphor resistance protein CrcB
	spdC, SAOUHSC_02611	Regulates walKR two-component system. Involved in antimicrobial resistance, biofilm formation.
C. jejuni	*tonB3*	Siderophore required for virulence
	htrB	Lipid A synthesis, heat shock, oxidative stress, osmotic stress, low pH.
	cdtABC	Core toxicity pathway proteins - cytolethal distending toxin
	cdtR	Binary toxin (CDT) regulator
	tcdC	Anti-sigma factor, regulates tcdAB endotoxin genes. tcdC mutations can trigger hypervirulence.
	cmeA	periplasmic fusion protein CmeA (multidrug efflux system CmeABC)
	cmeD	outer membrane component of efflux system (multidrug efflux system CmeDEF)
	cmeF	integral membrane component of efflux system (multidrug efflux system CmeDEF)
	cmeB	inner membrane efflux transporter CmeB (multidrug efflux system CmeABC)
	cmeC	outer membrane channel protein CmeC (multidrug efflux system CmeABC)
	Cj1174	putative efflux protein (multidrug resistance protein)
	Cj1375	putative multidrug efflux transporter
	Cj1173	putative efflux protein (multidrug resistance protein)

Supplementary Table 5. Selected head-on virulence, virulence related, and antibiotic resitance genes for each species.

Species	Total #	Genbank or Refseq Accession
Mycoplasma gallisepticum	11	NC_004829
		CP001872
		CP001873
		CP003506
		CP003507
		CP003508
		CP003509
		CP003510
		CP003511
		CP003512
		CP003513
Staphylococcus aureus	13	NC_007795
		AM990992
		CP012409
		CP006838
		CP010526
		AP009351
		CP000730
		FN433596
		CP002120
		CP002111
		CP002643
		CP003033
		HE579073
Campylobacter jejuni	21	NC_002163
		CP010463
		CP010483
		CP010494
		CP010498
		CP010511
		CP010484
		CP007190
		CP007191
		CP017229
		CP010492
		CP010480
		CP010475
		CP007193
		CP010471
		CP010906
		CP010307
		CP006851
		CP001876
		CP006688
		CP003871
Klebsiella pneumoniae	12	NC_016845
		CP000964
		CP011980
		CP006918
		CP018719
		CP016811
		CP020061
		CP014004
		CP015753
		CP015990
		CP015025
		CP016811
Bacillus subtilis	17	NC_000964
		CP002183
		CP016894
		CP017314
		CP017676
		CP018173
		CP018184
		CP017763
		CP002468
		CP003783
		CP009796
		CP011051
		CP011534
		CP011882
		CP014471
		CP014858
Mycobacterium tuberculosis		NC_018143.2
		CP001641.1
		CP003233.1
		CP003234.1
		CP004886.1
		CP005386.1
		CP010329.1
		CP010335.1
		CP010336.1
		CP010338.1
		CP010340.1
		CP010873.1
		NC_000962.3
		NC_002755.2
		NC_009525.1
		NC_009565.1
		NC_012943.1
		NC_016768.1
		NC_017522.1
		NC_017524.1
		NC_018078.1
		NC_020089.1
		NC_020559.1
		NC_021054.1
		NC_021194.1
		NC_021251.1
		NC_021740.1
		NC_022350.1
		NC_025025.1
		NZ_AP014573.1
		NZ_CP002871.1
		NZ_CP002882.1
		NZ_CP002883.1
		NZ_CP002885.1
		NZ_CP007027.1
		NZ_CP007803.1
		NZ_CP007809.1
		NZ_CP009100.1
		NZ_CP009101.1
		NZ_CP009426.1
		NZ_CP009427.1
		NZ_CP009480.1
		NZ_CP010330.1
		NZ_CP010337.1
		NZ_CP010339.1
		NZ_CP011510.1
		NZ_CP012090.1
		NZ_CP012506.2
		NZ_CP013475.1
		NZ_CP016794.1
		NZ_CP016888.1
		NZ_CP016972.1
		NZ_CP017593.1
		NZ_CP017594.1
		NZ_CP017595.1
		NZ_CP017596.1
		NZ_CP017597.1
		NZ_CP017598.1
		NZ_CP017920.1
		NZ_CP018300.1
		NZ_CP018301.1
		NZ_CP018302.1
		NZ_CP018303.1
		NZ_CP018304.1
		NZ_CP018305.1
		NZ_CP018778.1
		NZ_CP020381.2
		NZ_CP022014.1
		NZ_HG813240.1

Supplementary Table 6. Fully assembled genomes used in whole genome mutational analyses. Reference genome accession numbers are listed first for each species.

Script Name	Function
TZmap1.py	Processes TimeZone_v1.exe output data. Used for inverted gene identification from closed genomes. The outfile must then be analyzed using TZmap2.py.
TZmap2_v3.py	Processes TZmap1.py outfile to detect inverted genes from closed genomes. Outputs a spread sheet file.
multi_g2m_v12.py	Converts Genbank formatted whole genome files to a MochiView formatted flat file (similar to GFF3 format).
multi_allgeneGCskew_v6.py	Calculates GC skew values for all genes in all genomes in the target folder.
multi_add_ori_v3.py	Annotates the orientation of each gene in a MochiView formatted flat file.

Supplementary Table 7. Scripts used for GC skew and closed genome-based gene inversion analyses
