# Supplementary material for: Gene inversion potentiates bacterial evolvability and virulence
Source: Nat Commun. 2018 Nov 7;9:4662. doi: 10.1038/s41467-018-07110-3 (PMC6220195; doi:10.1038/s41467-018-07110-3)
Supplement: Supplementary file 3 — Description of Additional Supplementary Files [file 41467_2018_7110_MOESM3_ESM.pdf]

Supplemental Data.

Inverted alleles identified by whole-closed-genome analysis. The genes for which an inverted allele was identified in a particular lineage among 54 closed Mtb genomes are presented. Gene orientation and GC skew data are shown for each allele (either the inverted alleles or the non-inverted alleles). Data were collected and combined as follows: Homologous genes from each strain were identified by reciprocal blast using TimeZone software; gene orientation was identified using Genbank-formatted genome data which shows the location of each gene in the genome and coding strand; the replication origin/termination sites were identified by calculating the GC skew inflection points along the chromosome for each isolate; GC skew values were calculated for each gene region's leading strand using custom Python scripts. Data were combined using custom Python scripts. For cases in which a clear homologue was not identified in a given lineage, the cell was left blank. Notably multiple genes encoding Esat-6 (esxP, esxJ, and esxW) homologues identified as putative inverted alleles were thrown out because their highly similar sequences precluded accurate identification of the corresponding homologue. Python scripts are listed in Supplemental Table 7.
